# Secondary Somatic Embryogenesis in Plants: From Cellular Mechanisms to Biotechnological Potential

**DOI:** 10.3390/plants14223413

**Published:** 2025-11-07

**Authors:** Milica D. Bogdanović, Katarina B. Ćuković

**Affiliations:** Institute for Biological Research “Siniša Stanković”-National Institute of Republic of Serbia, University of Belgrade, Bul. despota Stefana 142, 11108 Belgrade, Serbia; katarina.cukovic@ibiss.bg.ac.rs

**Keywords:** recurrent somatic embryogenesis, repetitive somatic embryogenesis, cyclic somatic embryogenesis, direct SE, indirect SE, somaclonal variation, unicellular origin, multicellular origin, malformed SEs

## Abstract

Secondary somatic embryogenesis (SSE) is a powerful tool in plant biotechnology, enabling the continuous production of embryos from primary somatic embryos (PSEs) and offering broad applications across agriculture, forestry, horticulture, and pharmaceutical industries. Depending on culture conditions, SSE may proceed directly from the surface of PSEs or indirectly via callus formation, with the outcome strongly influenced by exogenous plant growth regulators (PGRs). A key advantage of SSE is its cyclic nature, which offers a valuable strategy to maintain embryogenic potential over extended culture periods, generating true-to-type embryos without reliance on the original explant, while significantly increasing the multiplication rate, often making SSE more productive than PSE in many species. This review explores in detail the cellular origin and developmental pathways of secondary embryos, the maintenance of embryogenic competence through cyclic embryogenesis, as well as genetic and epigenetic aspects and the biotechnological applications of this process. Moreover, it addresses challenges regarding strong genotype dependence, variability in embryo quality and morphology, limitations in maturation and conversion potential, and the gradual decline of embryogenic competence with successive cycles, all of which need to be overcome to ensure the stability and reproducibility of SSE and maximize its impact.

## 1. Secondary Somatic Embryogenesis: Principles and Applications

The discovery of plant cell totipotency, which revealed that certain cells have the capacity to differentiate into a fully developed organism [1,2], laid the foundation for the concept of somatic embryogenesis (SE), which today has a pivotal role in the plant biotechnology toolbox, offering a powerful method for clonal propagation and genetic manipulation [3]. In this process, also known as primary somatic embryogenesis (PSE), somatic cells produce embryogenic tissue under inductive conditions, resulting in somatic embryos (SEs) [4]. These primary somatic embryos (PSEs) can develop into complete plants or initiate another cycle of SE, which is known as secondary somatic embryogenesis (SSE), cyclic, recurrent, or repetitive embryogenesis [5]. This cyclic nature provides a continuous source of embryogenic tissue for various downstream applications, like mass plant propagation, crop improvement, and germplasm conservation [5,6].

SSE is influenced by many factors, mainly the type and concentration of plant growth regulators (PGRs) and nutrient media components, physical culture conditions, as well as primary explant type. The most commonly reported auxin for SSE induction is 2,4-dichlorophenoxyacetic acid (2,4-D), often applied in synergy with cytokinins that primarily contribute to cell differentiation and embryo proliferation. The combination and concentrations of PGRs affect both the success of SSE induction and normal embryo development. Environmental factors such as temperature, light, and culture medium composition further determine embryogenic competence and can sometimes influence the outcome of embryogenesis. The success of SSE also largely depends on the tissue used as the primary explant for SSE induction. Whole PSEs generally possess a high capacity for inducing SSE and are therefore often used as starting material. Since these conditions are often highly species- and even genotype-specific, continuous optimization and molecular research are required to improve protocol standardization and applicability to a wide range of species relevant for various industries, such as agriculture, forestry, horticulture, and pharmacy.

The developmental pathway of SEs can be direct, where new embryos form directly from single cells or small cell groups on the explant surface without an intervening callus phase, or indirect, involving an intermediate embryogenic callus stage. Histological studies report both unicellular and multicellular origins of secondary somatic embryos (SSEs), often emerging from epidermal or sub-epidermal cells. Unicellular origin has been linked to better embryo development [7], offering advantages for clonal propagation and genetic transformation [8], where preserving genetic identity is of great importance. Like PSEs, SSEs often progress through typical developmental stages: globular, heart-shaped, torpedo, and cotyledonary, and generally lack a vascular connection to the parent tissue [9]. Their development is often asynchronous, with multiple stages present simultaneously on a single PSE [5,10,11].

A major benefit of SSE is its cyclic nature, allowing for continuous and iterated production of embryos over extended periods without reliance on the original explant. This repetitiveness significantly boosts the multiplication rate, making it more efficient than PSE in many species. In some cases, this can result in the production of an exceptionally large number of SSEs, effectively regenerating 20^13^–20^26^ SSEs in a year [12] and 74,000 SSEs after eight subculture periods [13], from a single PSE, as reported for *Manihot esculenta* Crantz and *Lepidosperma drummondii* Benth., respectively. Although SSE provides a tool for effective plant multiplication, repeated cell divisions can pose risks for genome and epigenome instability, often leading to somaclonal variation [14,15]. This can also sometimes result in SEs with malformed structures, which can negatively affect germination and conversion to plants.

Applications of SSE in plant biotechnology are extensive. High multiplication rates, due to the cyclic nature, make SSE an ideal method for large-scale plant multiplication, while the single-cell origin of many SSEs enables more predictable and reliable results in genetic transformation, reducing the likelihood of chimeric plants [6]. For elite lines, and rare and endangered plants, SSE provides a suitable method for germplasm conservation, allowing the long-term storage and maintenance of embryogenic cultures. Furthermore, it serves as a valuable research model for studying plant cell differentiation and developmental pathways, broadening the essential concepts within plant fundamental biology [5].

Although more than 130 review articles indexed in Scopus can be found covering PSE, with focus varying from developmental aspects of SE [16] to biotechnological applications [17], SSE has been systematically reviewed in only one review article so far, published in 1995 [6]. Due to the accumulation of new studies researching SSE explicitly in different species, there is a high-priority need for a new comprehensive overview of the available literature, especially having in mind a broad range of biotechnological applications. The cyclic nature of SSE, which is clearly distinguished from PSE, makes it even more attractive for potential applications, highlighting the need to specifically address the repetitiveness of the process in literature overviews. This review will examine in detail cellular and developmental processes that govern the induction and cyclic nature of SSE, genetic and epigenetic considerations, and a wide range of applications within the field of plant biotechnology. Remaining challenges and future research directions in this prosperous domain will also be discussed.

To provide a comprehensive overview of the existing literature, an extensive Scopus document search was performed, using several terms commonly used to describe SSE: “secondary”, “cyclic”, “recurrent”, and “repetitive” in combination with “somatic embryogenesis”, limited to journal articles and book chapters. After manual curation, a list of 546 scientific articles related to SSE and published between 1983 and 2024 was formed, with query terms in fields: article title, abstract, and keywords. Among these, 133 studies were narrowly focused on SSE (https://hdl.handle.net/21.15107/rcub_ibiss_7767 (accessed on 6 November 2025)), containing search terms in the article title indicating that these studies were covering SSE in great detail, rather than mentioning it without additional information. These studies, consisting of 1 review article, 1 book section, and 131 research articles, served as the primary literature sources used in this manuscript. A wide range of plant species (85 species) was studied, many of which are economically important for industries such as agriculture, wood and rubber production, horticulture, and medicinal and ornamental applications.

## 2. Cellular Origin and Developmental Pathways

### 2.1. Direct vs. Indirect SE

The initiation of secondary SE can follow either a direct or indirect pathway. Direct secondary embryogenesis is characterized by the formation of new embryos directly from the surface of pre-existing somatic embryos. This mechanism has been observed in various plant species, for example, in *Panax ginseng* C.A.Mey., where SSEs originated directly from the surface of PSEs on hormone-free MS (Murashige and Skoog) medium, exhibiting further proliferation in a cyclic manner [18]. In contrast, indirect SSE involves the formation of an embryogenic callus from the initial somatic embryo, which then gives rise to secondary embryos. In *Theobroma cacao* L., for example, both primary and secondary SE proceeded by the indirect pathway, where embryogenic callus preceded the formation of somatic embryos [19]. Some species can undergo both pathways, like *Centaurium erythraea* Rafn, which exhibits both direct and indirect secondary SE depending on the hormonal conditions [5]. Direct SSE is the more prevalent pathway in the literature, as identified in 44 studies, while the indirect pathway of SSE was described in 16, and both pathways were present in 18 articles. In general, the onset of SE by a certain pathway is determined by the physiological and morphological characteristics of the primary explant, as well as by the growth regulators used.

SEs obtained by direct embryogenesis are typically less numerous than SEs obtained by indirect embryogenesis [20], and direct SSE has been reported to be less frequent than indirect SSE [21]. However, direct SSE has some advantages over indirect SSE, mainly related to the absence of a callus phase, which is noted to be a significant source of somaclonal variation, genomic alteration, albinism, and sterility [20,22]. With direct SSE, chances for genetic variation are lower in the progenies obtained by this pathway, making the method very useful for genetic transformation experiments [23]. Direct SSE can also yield embryos faster, as it generally requires less cell reprogramming than indirect SE [20]. This could be due to the effect that proembryogenic competent cells are already present in the explant [5], and that these cells, capable of direct SE, are physiologically similar to those in zygotic embryos, and therefore easily proceed to the direct pathway, often without the need of PGRs [6]. On the other hand, indirect SSE was often noted to be more proliferative and can develop embryos faster than direct SSE, like in *Dianthus caryophyllus* L. for both precotyledonary and cotyledonary PSE explant types [24]. Therefore, indirect SSE is generally the preferred pathway for large-scale propagation [20], although it often requires the use of PGRs [6].

The choice of medium and PGRs used often determines whether the SSE pathway occurs directly or indirectly. For example, in *Commiphora wightii* (Arn.) Bhandari, full- and half-strength CIM (callus induction medium) and half-strength SH (Schenk and Hildebrandt) medium gave rise to direct SSEs on the cotyledon and hypocotyl tips of primary explants, while full-strength SH media favored the indirect pathway with a callus phase [25]. In *D. caryophyllus*, the presence of 2,4-D enabled both direct and indirect SSE pathways; however, on media containing 6-Benzyladenine (BA) or benzylaminopurine (BAP), only indirect SSE was observed, and interestingly, direct SSE only occurred once, whereas indirect SSE was observed repeatedly [24]. When picloram was used in the same species, the type of primary explant determined the pathway, with precotyledonary PSEs giving rise to SSEs directly and cotyledonary PSEs indirectly [26]. Interestingly, in some cases, even the genotype background determined the developmental pathway, as in the case of *Medicago truncatula* subsp. *narbonensis*, where all tested genotypes formed SSEs directly, except genotype C7, which formed the embryogenic callus and subsequently SSEs by the indirect pathway [27]. In *Quercus robur* L., on PGR-free medium, direct SSE was the exclusive response, while on media with PGRs, both pathways occurred, depending on the hormone used, with 1-naphthaleneacetic acid (NAA) favoring the direct and indole-3-butyric acid (IBA)/BAP combination the indirect pathway [28]. In *C. erythraea*, direct SSE was the exclusive pathway on PGR-free and 2,4-D-only media, but also the predominant pathway on media containing both 2,4-D and synthetic cytokinin N-(2-chloro-4-pyridyl)-N’-phenylurea (CPPU) [5]. Although PGRs are usually considered the main inducers of the developmental pathway, this is not always the case. In *Q. robur*, for example, the balance between direct and indirect SE was primarily determined by the embryogenic competence of epidermal cells [28]. Regardless of the PGRs used, explants derived from small translucent embryonic structures exhibited a higher embryogenic capacity and more frequently produced direct SE than explants from more advanced structures, indicating that the ability for SE depends more on the undifferentiated state of the tissue than on the influence of PGRs applied [28].

The relation between hormones applied and the pathway realized is nevertheless a complex one. Different tissues no doubt exhibit a different potential to enter SSE, probably due to the internal hormone levels, gene expression levels, and other predetermined conditions, which affect how this tissue will respond to wounding or PGR exposure during SSE. In general, PGR exposure induces more cellular stress and reprogramming, resulting in callus formation and the indirect pathway [29], even in the case of more differentiated and less embryogenic-competent tissues like leaves. On the other hand, a direct pathway usually requires the presence of proembryogenic competent cells, physiologically more similar to zygotic cells in terms of gene expression and internal hormone cues, which are probably present in abundance in primary SSEs, and which can readily enter direct SSE even without external PGRs. This was reported in 19 studies, where the direct pathway of SSE correlated with SSE media without PGRs; however, 25 studies reported the direct pathway in combination with PGRs, suggesting a more complex relationship. On the other hand, the indirect pathway correlated with PGRs present in the SSE media in 15 articles, while only 1 study reported SSE without PGRs, indicating a strong need for PGRs during indirect pathway expression.

Apart from the presence or absence of callus, a detailed histological analysis is helpful for distinguishing between these two pathways, although very few studies describe in detail this difference in SSE. As reported in Zegzouti et al. [28] on *Quercus robur* L., in the indirect pathway, the cortical parenchyma cells of primary explants displayed the early signs of histological modification, followed by the formation of calli composed of vacuolated cells and clumps of densely stained, mitotically active cells underneath the explant surface [28]. The resulting embryos forming from the calli lacked provascular tissues and did not show any starch or protein body accumulation. In the direct pathway, SSE originated exclusively from epidermal cells, which divided periclinally instead of following the normal anticlinal orientation, suggesting the presence of pre-embryogenic determined cells in the epidermis expressing a new developmental pathway [28]. The SSEs were then initiated by the formation of spherical globules delimited by the epidermal layer, which further developed into typical embryos. Similarly, direct SSEs in *Sorbus pohuashanensis* (Hance) Hedl. arose from the rapid division and proliferation of primary SE epidermal cells, following the typical morphology and SSE developmental stages [30]. Alternatively, both direct and indirect SSEs originated from the subepidermal cells of primary explants, as described by Bogdanović et al. [5] for *C. erythraea*. The pathway was initiated indirectly, from the proembryogenic masses formed in the callus, or directly, following the activation of repeated cell divisions of the proembryogenic cells. Indirect SSE initiated the formation of embryogenic callus, comprisedof small and isodiametric clumps of cells, containing prominent nuclei and dense cytoplasm, and whose superficial layers exhibited meristematic characteristics [5].

### 2.2. Cellular Origin of SSE

In most cases (31 studies), SSEs formation is observed on the surface of primary explants. When a polarized explant such as primary somatic embryo is used, new SSEs can often be observed in certain zones of the PSE, like in the case of *C. erythraea*, where new embryos were usually formed directly in the hypocotyl zone on PGR-free media [5]. Where indicated, SSEs were formed most often on hypocotyls (10) [5,23,31,32,33,34,35,36,37,38], cotyledons (5) [39,40,41,42,43], at the base of PSE (3) [44,45,46], on the root pole (2) [47,48], or a combination of these organs, for example, on cotyledons and hypocotyls (5) [8,24,25,49,50], as well as hypocotyl-root zone (4) [18,51,52,53], cotyledon and root zone (4) [11,21,30,54], or other combinations.

Histological analyses were performed to determine the origin of SSEs within primary tissues. Epidermis has been listed as the most common source of proembryogenic cells (8) [8,11,28,30,40,41,46,55], but subepidermal [5], procambium [56], or perivascular layers [57] are also reported, as well as combinations of epidermal and subepidermal [33,43,58,59], epidermal and perivascular [60], or mesophyll [61]. In an exceptional case, fully differentiated stomatal guard cells have the ability to directly form SSEs in *Oncidium* orchids, consisting of two enlarged cells originating from guard cells [61].

Histological analyses also provide invaluable insights into the cellular origins of SSEs, which vary between species, with some studies suggesting a multicellular origin while others indicate a unicellular origin. In *M. esculenta*, for example, SSEs arise from embryogenic masses that form around the procambium throughout the explant, which later fuse together to give rise to somatic embryos, suggesting a multicellular origin [56]. In *Bactris gasipaes* Kunth, epidermal and subepidermal cells, characterized by numerous small vacuoles, a large central nucleus with prominent nucleolus, no cell wall thickening, and plasmodesma, participated in the formation of multicellular SSEs [59]. Similarly, subepidermal cells of primary SEs gave rise to SSEs with a multicellular origin in *C. erythraea* [5].

A unicellular SSE origin has been noted to be important for transformation though repetitive embryogenesis [8], where if it is possible to obtain a somatic embryo from a single transformed cell, secondary embryogenesis could be used to increase the number of transgenic embryos prior to plant recovery, while in the case of multicellular SSE origin, chimeric transgenic sectors are often obtained, and repetitive SE under selection is needed to reduce non-transgenic tissues [26]. This was evidenced in *Juglans regia* L., where the four-cell SSE structure was seen to be derived from a single surface cell where transverse to oblique divisions produced the pro-embryo structure [8]. Although there was also histological evidence of anticlinal non-embryogenic divisions of surface cells, they were different from embryogenic divisions based on the appearance of the derivative cells—the non-embryogenic derivative cells were highly vacuolated and separated by a clearly differentiated cell wall, while embryogenic cells were more densely cytoplasmic [8]. In *Hevea brasiliensis* (Willd. ex A.Juss.) Müll.Arg., histological observation found that SSEs were mainly originated from single epidermal cells undergoing unequal periclinal divisions, which first produced a two-celled pro-embryo and later a multicellular pro-embryo [43]. While primary SEs arise from clusters of cells formed on staminoids in *T. cacao*, secondary SEs arise predominantly from the division of single cells in cotyledons of primary embryos, and thus have a unicellular origin, often observed to be linked to proper embryo development [7].

The contrasting cellular origins and developmental pathways observed across different plant species highlight the diversity of mechanisms underlying SSE and the need for species-specific optimization of culture conditions.

### 2.3. Understanding and Managing Abnormal Morphology of SEs

Abnormal, malformed SEs are observed in both PSE and SSE across various plant species. These abnormalities include a wide range of morphological deformations, most often related to the number, shape, and organization of cotyledons and the hypocotyl. They are often attributed to factors such as inappropriate PGR concentrations, the long-term maintenance of cultures, the specific medium composition, and a multicellular origin of SEs.

Some form of malformed SEs was observed in 23 studies narrowly focused on secondary SE. Common types include embryos with a single, three, or multiple cotyledons, fused cotyledons, trumpet or plate-like shapes, as well as fasciated embryos formed by the fusion of several embryos [5,7,10,30,31,62]. Some embryos show a fused, thickened, reduced, or completely absent hypocotyl [36,63,64]. Abnormal SSEs can also appear hyperhydric, yellowish, and milky or even relapse into the callus phase [20]. In many cases, such embryos with an altered morphology fail to germinate, especially under suboptimal nutrition [36,65], or convert into malformed plantlets [31].

**Figure 1:**
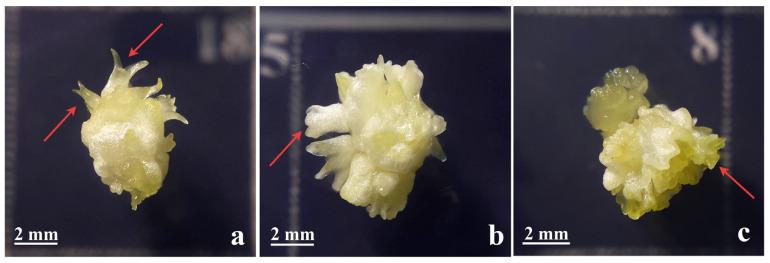
Appearance of normal (**a**) and abnormal SSEs with multiple cotyledons (**b**) or fasciated morphology (**c**), as indicated by red arrows. The figure was modified from Bogdanović et al. [5]. SSEs–secondary somatic embryos.

The most common causes of morphological abnormalities in SSEs are related to an inadequate culture medium composition, especially the type, concentration, and prolonged use of PGRs. The most notable culprit is the synthetic auxin 2,4-D, which, in higher concentrations, often leads to malformed embryos with multiple or fused cotyledons, trumpet-shaped structures, and fasciation (Figure 1), as shown in *C. erythraea* by Bogdanović et al. [5], where a higher concentration of 2,4-D and CPPU correlated with a higher incidence of abnormal SSEs. 2,4-D can disrupt cell polarity and lead to excessive cell division, impairing the spatial coordination needed for normal embryo development [66]. As an alternative, Daigny et al. [40] suggested that NAA supports a better embryo morphology than 2,4-D. Zegzouti et al. [28] confirmed this, showing that NAA promotes direct SSE with well-formed embryos in *Q. robur*, while IBA/BAP combinations induced indirect pathways associated with poorly structured embryos, showing a lack of procambial strands, no starch or protein storage, with a wide basal connection to callus tissue.

In addition to excessive or imbalanced PGRs, several studies reported that long-term maintenance on PGR-containing media promotes the frequent occurrence of abnormal structures [11,30,62]. For example, long-term maintenance on PGR-containing medium in *C. camphora* resulted in about 9.25% anomalous SSEs, of which 5.96% were embryogenic clusters [11]. A similar trend was noted in other species, such as *Albizia lebbeck* (L.) Benth. and *S. pohuashanensis*, where a prolonged culture on PGR-containing media yielded embryos with either single or multiple cotyledons or other abnormalities, such as trumpet-shaped embryos with fused cotyledons [30,62]. The subculturing frequency is also sometimes relevant, as shown for *Prunus persica* L., where most embryos became abnormal, with fused cotyledonary lobes and disorganized growth, during 40 days of cultivation. On the other hand, subculturing after 10 days resulted in poorly developed, small SSEs arrested in globular and heart-shaped stage [55].

The carbohydrate source is another factor to consider for maintaining proper embryo development. For example, Bao et al. [65] reported that 30 or 60 g/L sucrose led to 50% abnormal embryos in *Rosa hybrida* E.H.L. Krause, while using same concentrations of glucose significantly reduced the number of abnormalities. Similarly, Śliwińska et al. [34] found that a high concentration of sucrose (30 g/L) caused cotyledon fusion and twisting in *Polyscias filicifolia* (C. Moore ex E. Fourn.) L. H. Bailey, whereas lowering it to 15 g/L eliminated these defects.

In some cases, the developmental maturity of embryos was a key factor. Several authors pointed out that abnormalities such as fused hypocotyls, the absence of roots, or cotyledons often occur because embryos fail to reach full morphological and physiological maturity [36,64]. As a solution, Yadollahi et al. recommended using ABA and PEG, which synergistically promote proper SSE maturation, prevent precocious germination, and lead to the formation of normal torpedo and cotyledonary-stage embryos in *Brassica napus* L. [36]. In some cases, embryo malformations were linked to their multicellular origin. The larger number of abnormal embryos produced during primary SE, which were multicellular in origin, could be due to the variation in the number of cells that participate in the formation of each individual, as well as the possible lack of effective spatial coordination during development. On the contrary, higher embryo conformity in terms of a lower number of abnormal embryos, observed in *T. cacao*, was related to a unicellular origin [7].

Physiological and morphological abnormalities in embryo development can be important challenges for reliable propagation. As previously discussed, such abnormalities often impair embryo germination and successful conversion into plantlets. One possible strategy to mitigate this limitation is recycling through SSE, which can yield new embryos with a normal morphology [67]. Other proposed solutions often involve optimizing hormone ratios and medium formulations, or incorporating additives like ABA or PEG, to promote normal development and maturation.

## 3. Cyclic Embryogenesis-Maintaining Embryogenic Potential

**Figure 2:**
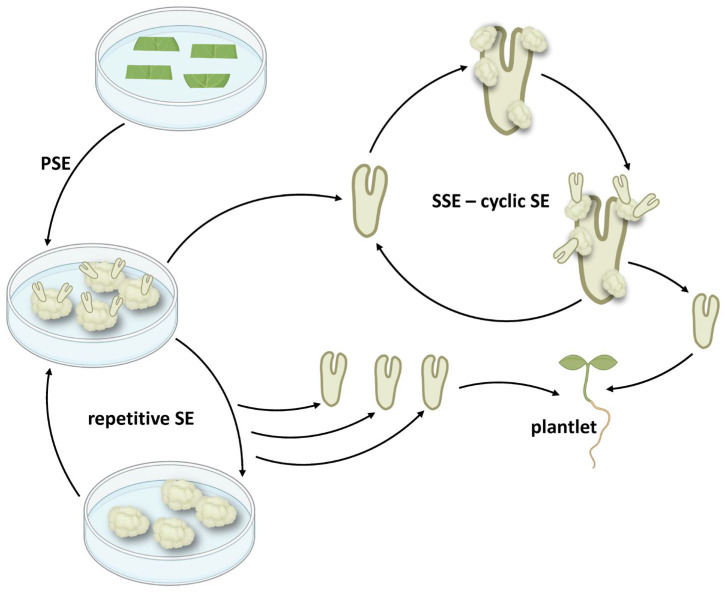
Differences between cyclic and repetitive SE. For both pathways, primary embryos are obtained by PSE from leaf explants of in vitro plants. PSEs can then be used for the initiation of SSE and subsequent cycles (cyclic SE), or PSE tissues can be maintained by non-selective subculture (repetitive SE). Both pathways can be used as a source of new SEs that can germinate into plantlets. SE—somatic embryogenesis; PSE—primary somatic embryogenesis; SSE—secondary somatic embryogenesis; PSEs—primary somatic embryos.

When the embryogenic potential can be maintained for extended periods through repeated cycles of SE on embryos from previous cycles, the production of SEs can be greatly enhanced and prolonged, without depending on the original explant. In cyclic SE, SSEs isolated in one cycle become the explant for the next SE cycle and so on (Figure 2). Due to generally higher multiplication rate than other regeneration systems, cyclic SE is therefore an attractive method for massive clonal propagation and is especially advantageous for gene editing and genetic transformation applications because it produces true-to-type SEs [20]. This should possibly be distinguished from repetitive SSE obtained by several subcultures of PSE structures, such as primary embryogenic callus [68,69,70,71,72], PSE clusters [13], or cultures obtained from PSEs [73], where new embryogenic structures are generated repeatedly from a non-selective subculture of primary tissues (Figure 2). Repeated generation of SSEs from PSEs was discussed as the lack of some internal controls that exist in the intact plant [73]. In many species, since embryogenic competence in culture generally declines over time due to factors like aging and prolonged subculturing over several months, cyclic SE is often used as a method to restore or maintain the embryogenic potential of important productive lines.

Cyclic embryogenesis and long-term maintenance of the embryogenic potential through subculturing of SEs have been reported in 40 studies. While most articles report the maintenance of embryogenic potential during 1–3 years (13), in some cases, such as *Vitis rupestris* Scheele, regeneration by cyclic SSE was reported for up to 10 years [74]. The number of reported cycles ranged from two to twelve (17), most commonly between two and five (11). However, factors affecting the maintenance of embryogenic potential in subsequent cycles are still largely unknown and highly variable among different species.

In many cases, the embryogenic potential was reduced during cultivation in repeated cycles of SE. In *T. cacao*, for example, the number of quaternary SEs was lower than the number of tertiary SEs, leading to a diminishing capacity for embryogenesis through three successive cycles [20]. Similarly, in *D. caryopyllus*, no significant differences were detected in the percentage of explants undergoing cyclic SE, but the number of SEs induced on each of the explants was reduced significantly with increasing cycles [26]. In cabbage (*Brassica oleracea* L. var. *capitata*) and cauliflower (*Brassica oleracea* L. var. *botrytis*), the embryogenic potential of the cultures was maintained for 10 months (ten cycles) but with a reduction in the frequency through the cycles, more pronounced after the eighth subculture: for cabbage, it was reduced more drastically, from 83.3 to 34.2%, and for cauliflower, it was reduced from 87.5 to 51% [53]. In *Akebia trifoliata* (Thunb.) Koidz., even though cyclic SE could progress through seven cycles, the capacity for SE induction and the mean number of SEs per explant, as well as the mean fresh weight of SSEs, declined with the recurrent cycles [48]. Although reasons for this decline in SE potential are still largely unknown, the authors attribute this decline to factors like somaclonal variation causing genetic or epigenetic variation, dysfunction or loss of genetic regulatory mechanisms, altered gene expression, reduction in the number of cells competent to undergo SE, nutritional limitations, or changes in hormonal balance or sensitivity to exogenous PGRs [5,20,48,64].

In other cases, the embryogenic potential was successfully maintained over the long cultivation period [40,42,60,74,75,76,77], even in cases with PGR-free media [31], or alternatively even increased, at least for some genotypes [44,78,79]. Markedly, in *M. esculenta*, across the three obtained cycles, production more than doubled from 7.5 embryos per initial embryo in the first cycle, to 18.6 and 20.8 in the second and third cycles, respectively [80]. For the same species, the authors mentioned obtaining more than 20 new mature SEs in 2–4 weeks, resulting in 20^13^–20^26^ mature SEs a year, in a well-established system [12]. In *A. lebbeck*, embryogenic lines were maintained for about three years with long-term persistence of embryogenic competence, with the number of differentiating embryoids increased up to the fourth subculture, after which a nearly constant number of regenerated embryoids was observed [62].

Embryogenic response in cyclic SE, characterized by embryogenic callus production, can be affected differently from SE development, especially the development of cotyledonary SEs. This was illustrated in the case of *C. erythraea*, where the production of embryogenic callus increased in subsequent cycles when cotyledonary SEs were subcultured, but the production of cotyledonary SEs decreased, suggesting that although the embryogenic potential of the explants did not decrease, the rate of the embryo maturation slowed down with subculturing, probably due to long-term exposure to 2,4-D, which ultimately affected the maturation of SEs [5]. Lower concentration of PGRs, for example, 2,4-D and CPPU, in the same species can promote more cycles and effectively produce more cotyledonary SEs, but even the low concentration of 2,4-D of 0.1 mg/L apparently delayed the maturation of newly produced SEs [5]. In a similar case, continual exposure to higher concentrations of 2,4-D (30 or 40 mg/L) led to excessive nonembryogenic callus formation in *Arachis hypogaea* L., which negatively impacted both primary and repetitive embryogenesis, which were successful on concentrations of 10 or 20 mg/L [81].

While the majority of tested species could undergo cyclic SE by subculturing SSEs, there are also some exceptions. When PSE in *Brassica campestris* L. was induced on media with BAP and then transferred to media without PGRs for SSE formation, subculturing SSEs on the same media without PGRs did not proliferate further but developed into shoots, with the authors speculating that the secondary response was a carry-over effect of the primary treatment [49].

In *M. truncatula*, the ability to maintain cyclic SE potential seemed to be dependent on the genotype, and out of one genotype of the subspecies Narbonensis (SLA) and four genotypes of the Jemalong cultivar (N1, M5, M9, and C7), only three genotypes (SLA, M9, and N1) produced cyclic SSE, in the duration of 2 years for the SLA genotype and for more than 1 year for genotypes N1 and M9 [27]. In *M. sativa*, different genotypes were consistent in the number of new SEs produced by the monthly cycles, with genotype TE26 producing an average of 20 SEs per embryo, while TE66 produced 30 [76]. Culturing conditions have also been reported to affect cyclic culture, for example, in *B. gasipaes*, where efficient cycling cultures could only be established on a temporary immersion system (TIS), while on a solid culture medium, the embryogenic potential decreased rapidly, to only 2% after four cycles [59].

There is a large variation in the time and number of cycles that an embryogenic culture can sustain, but the underlying causes for this variation are still unknown. Physiological deterioration or genetic instability accumulated in culture over time could lead to a loss of embryogenic potential and thus a finite number of cycles. If the initial number of secondary embryos was low, the potential for propagating many subsequent cycles from that limited initial pool would also be reduced. The same is true for cases with a declining number of available explants of a certain development stage (e.g., cotyledonary embryos) needed for the next cycle [5]. Factors contributing to long-term maintenance of embryogenic culture seem to be a genetic predisposition (finding the right genotype with the ability to maintain embryogenic potential) [27,51,76,82]; a well-optimized medium composition and PGR balance, unique to the species and sometimes even to the developmental stage of the explant [14,62]; and effective subculturing that utilizes the optimal developmental stage [14,83] to prevent aging of the culture, nutrient depletion, or accumulation of inhibitory metabolites (e.g., ethylene) [47,59]. For example, too long (e.g., more than 20 days) or too quick (e.g., 10 days) subculturing intervals can lead to abnormal embryo morphology or decreased multiplication rates [55]. In addition, the culture conditions should be optimized to steer away from maturation and conversion into plantlets since embryo generation will naturally cease once most embryos differentiate [6,84,85].

Despite the best efforts to maintain cultures, long-term recycling on media with PGRs can sometimes influence the higher frequency of malformed embryos [11,30,62], especially on higher PGR concentrations [5], effectively hindering the conversion of embryos into plants. However, cyclic SE could be a way to recycle those malformed embryos and obtain more germination-ready plants [67]. Long-term culture and exposure to PGRs has been known to cause somaclonal variation and genetic and epigenetic changes, which can affect the morphology and development of SEs [14,80,86,87].

Rather than being solely determined by culture conditions, some embryogenic cells may possess an inherent genetic program for cyclic embryogenesis, meaning that the ability to undergo SSE is a trait already present in the original plant. For example, parent plants and plants regenerated from somatic embryos of *Daucus carota* L. did not differ in their capacity to initiate repetitive SE from callus, suggesting that embryogenic cells derived from seed coats already possess a specific genetic program needed for SSE induction [52]. In another case, PSEs of a particular alfalfa genotype began to germinate prior to the initiation of RSE, indicating the presence of a distinct gene expression program, probably associated with endogenous hormone levels [76].

## 4. Genetic and Epigenetic Considerations: Stability and Variation

Genetic and epigenetic stability is an important consideration in both PSE and SSE, especially affecting the downstream application of SE-derived plants. While many studies have reported high genetic and phenotypic stability in plants regenerated through SSE [21,22,24,31,41,53,88,89], the potential for somaclonal variation cannot be ignored. Somaclonal variation, including genetic and epigenetic changes inducing obviousor hidden phenotypic variation, can arise with many in vitro culture techniques, especially the ones that involve the formation of intermediate callus tissue. Consequently, PSE, especially by the indirect pathway, can lead to increased genetic variation due to the serious genetic reprogramming required for the transition from somatic to embryogenic cells [5,15,22,41], while SSE tends to maintain the genetic stability of SEs derived from established embryogenic cultures, especially if occurring directly and from single cells [7,15,20,22,23,31,33,68,90,91,92]. In addition, long-term culture of embryogenic tissues can increase the risk of somaclonal variation, affecting the genetic stability of regenerated plants [14,15,21,43,60,86,87]. However, in *H. brasiliensis*, the rate of somaclonal variation was low in initial and final (8th–10th) multiplication cycles of SSE, with a temporary increase during the cultivation period, which indicated that the genome remained stable during multiplication and the total variation rate remained low [43]. Despite detected somaclonal variation, the chromosome number of the regenerated plants was similar to the mother tree, indicating chromosomal stability under the tested conditions, and the authors concluded that the multiplication method was adequate for commercial production [43]. The transition to SSE can act as a screen for mutation transmission, as PSEs carrying significant mutational loads may fail to further produce secondary embryos [41].

Genetic variations, that include changes in ploidy, chromosomal rearrangements, point mutations, and the insertion of mobile DNA elements or retroelements [43], and epigenetic modifications, such as DNA methylation and histone modifications, are likely to play a significant role in the generation of somaclonal variation in SE. In *Coffea arabica* L., for example, the main change observed in plants derived from embryogenic suspensions and SSE was aneuploidy: 7 of the 11 variant resulting plants had the loss of 1–3 chromosomes, while other somaclonal parameters, like phenotypic changes, AFLP and MSAP analyses for length, and methylation polymorphisms, showed very low variation rates [15]. In *T. cacao*, microsatellite variations observed in SSEs that were non-cryopreserved included allele loss, slippage, and generation of novel alleles, most likely due to small-scale events (insertions/deletions and point mutations) and rarely due to chromosome loss or large-scale deletions, while SSEs cryopreserved through encapsulation–dehydration revealed no polymorphisms, confirming the detrimental effect of long-term maintenance of embryogenic cultures on genetic fidelity [41]. DNA methylation and chromatin remodeling have been speculated to influence embryogenic competence [59,85], and are, in turn, influenced by auxin levels [15]. In terms of epigenetic modifications in SSEs, in *C. arabica*, molecular analysis showed that no resulting plant accumulated more than three methylation polymorphisms (ranging from one to three), indicating that neither SSE nor embryogenic suspension induced additional stress at the methylation level [15].

Understanding these mechanisms in the context of embryogenic competence and embryo development is crucial for improving the efficiency and reliability of SSE. This necessitates careful monitoring and the implementation of strategies to minimize somaclonal variation. Some strategies to overcome this risk could include the optimization of culture conditions to minimize stress-induced genetic and epigenetic changes, such as a reduced concentration or reduced time of exposure to high concentrations of PGRs [14,15,48,90], avoidance of the callus phase through the direct regeneration pathway [23,33,53,93], or the development of selection methods to identify genetically stable embryos.

## 5. Applications in Plant Biotechnology

The advantages of secondary and cyclic SE in plant biotechnology are well-documented, and their importance continues to grow. The plant biotechnology areas most benefiting from SSE are mass propagation and clonal multiplication of high-value plants, genetic transformation and crop improvement, and germplasm conservation and cryopreservation of endangered species. The non-chimeric nature, often single-cell origin, and genetic fidelity of plants derived from SEs are attractive traits of any multiplication and genetic transformation system. The capacity for continuous embryo production in repetitive and cyclic SSE systems provides a renewable and readily available source of plant material, especially valuable when the starting material is scarce. SE is also beneficial for species that are recalcitrant to other regeneration methods. Combined with the ability of SSE to enhance and prolong the embryogenic competence of specific lines, significantly increase the number of embryos produced [78], and recycle SEs of abnormal morphology [67], it is clear why SSE and cyclic SE are desirable methods in plant biotechnology. Because SSEs are characterized by a high multiplication index, repeatability, independence from explant source effects, and high level of uniformity, they are more reliable than other multiplication methods. In cases where embryogenic competence declines over time due to aging and subculturing effects, SSE could restore the embryogenic potential of lines maintained in vitro [5,78]. Economically important plants can benefit greatly from these methods, as maintaining genetic uniformity is paramount, while retaining a high multiplication rate. Since important cultivars are often recalcitrant to some of the propagation methods, or the methods are genotype-specific and inefficient, SSE contributes to diversifying the toolbox of available methods to promote propagation success.

SSE provides an efficient and scalable method for mass propagation and clonal multiplication of valuable plant species and elite genotypes and cultivars, generating a large number of genetically identical plants. This is particularly advantageous for species that are difficult to propagate by traditional methods (e.g., seeds, cuttings) or for the conservation of endangered plant species, where starting material is limited. SSE and cyclic SE significantly increased embryogenic potential and SE yields in Douglas fir (*Pseudotsuga menziesii* (Mirb.) Franco) compared to primary lines, even for the less productive genotypes [78,94]. In cassava (*M. esculenta*), the embryogenic frequency increased from 58% in PSE to up to 80% using SSE, accelerating embryo production to under 30 days and whole plant regeneration and hardening to just 4 months [39]. Repetitive embryogenic cultures of peanut (*A. hypogaea*) were maintained for over one year without apparent loss of embryogenic potential in liquid medium with exponential growth, enabling large-scale plant regeneration and conversion, while avoiding the continuous initiation of cultures from fresh explants [77]. For Moroccan cork oak (*Q. suber*), SSE with the addition of polyamines and ABA was a powerful tool for massive multiplication and maintenance of somatic embryos, saving time and effort compared to PSE [95,96]. In Asian ginseng (*P. ginseng*), SSE showed a higher ratio of embryo production than PSE, allowing large-scale propagation for prolonged periods by repeated cycles [18]. In *Narcissus* ‘Carlton’, a developed repetitive SE protocol yielded more than 20 embryos per 100 mg of callus (compared to 3.3–11 embryos from PSE), and it was efficiently maintained for over two years [69]. For *L. drummondii*, a species difficult to propagate by seed and cuttings but important for post-mining biodiversity restoration, a system producing over 74,000 SSEs from initial zygotic embryos after eight subculture periods (or 22,000–44,000 SEs per gram of embryogenic tissue) was developed [13]. A rapid (6 to 10 weeks) and repetitive SSE system was developed in sweet potato (*Ipomoea batatas* (L.) Lam.), allowing for prolific plant production and the efficient generation of large numbers of SSEs. These SSEs were often more numerous and synchronized than PSEs, allowing for easier germplasm conservation and gene transfer research in sweet potato [38].

SSE can also serve as a valuable tool for genetic transformation and crop improvement, either by classic genetic modification or by the increasingly popular genome editing, like the CRISPR/Cas9 system. The single-cell origin and non-chimeric nature of SSEs, especially regenerated directly, are attractive for gene transfer methods. This technique is especially valuable for vegetatively propagated crops, where conventional breeding methods are much less effective. In coffee (*C. arabica*), SSE aids in the improvement of genetic transformation protocols by electroporation of torpedo-stage PSEs, resulting in a significantly greater production of transgene-expressing SSEs compared to PSEs [23]. For alfalfa (*M. sativa*), a method for genetic transformation via *Agrobacterium tumefaciens* and the indirect SSE pathway was improved, with high efficiency (15.2%) and efficient kanamycin selection [97], building upon previous successful transformation results reporting a low transformation frequency but abundant secondary proliferation [98]. *R. hybrida* SEs have also been successfully transformed using *A. tumefaciens*, resulting in phosphinothricin-resistant regenerated plants [99]. Similarly, phosphinothricin-resistant transgenic cauliflower (*B. oleracea* var. *botrytis*) lines were obtained and found to be equal to non-transgenic plants in morphology and yield [100]. An efficient and reproducible *Agrobacterium*-mediated transformation system via repetitive SSE was developed for *Rosa rugosa* Thunb., achieving a high transformation efficiency of 11.4% when using somatic embryo clumps at the globular stage as explants [101]. Grape (*V. rupestris*) genetic modification has long benefitted from the use of SSEs as explants for transformation, owing the efficiency and reliability of the protocols to the participation of epidermal cells in callusing [74]. Similarly, in walnut (*J. regia*), superficial single cells give rise to SSEs, making SSE a suitable way to obtain fully transformed, non-chimeric plants [8]. In rubber tree (*H. brasiliensis*), SSEs were formed mainly from single epidermal cells of the cotyledons and with a cyclic nature of proliferation, which makes them a suitable target for *Agrobacterium* infection [43]. If, however, chimeric transformed embryos are obtained, repeated cycles of SSE under selective conditions can lead to the production of completely transformed embryos, thereby improving the efficiency and reliability of transformation procedures [6].

The maintenance of embryogenic cultures in vitro for extended periods provides a valuable resource for germplasm conservation. This approach is especially important for endangered species or those with limited seed viability. In the context of climate change and habitat loss, this is becoming particularly relevant, and the conservation of plant genetic resources, both wild and cultivated, is increasingly important. Cryopreservation techniques further enhance long-term storage and reduce the possibility of somaclonal variation [41]. For easier storage and transport, SSEs are a good source for artificial or synthetic seed production [25,26,63], although the cost-effectiveness of this method is probably more justified for hybrid crops like vegetables and flowers [6]. For cassava, SSEs were suitable for the mass propagation of disease-free elite clones and represented ideal candidates for germplasm conservation [102]. In mountain ash (*S. pohuashanensis*), which was subjected to interspecific competition and reducing regeneration capacity, efficient SSE could provide a sustainable source of plant material, contributing to large-scale vegetative propagation and germplasm resource conservation of this species [30]. SSE is routinely used for the long-term management of productive embryogenic lines of broadleaved tree species [94]. Moreover, in woody species characterized by long life cycles, preservation of embryogenic lines can be a cost-effective maintenance step until the lines have been tested in field conditions [6]. Cold storage of SSEs is suitable for germplasm preservation, arresting growth and metabolic processes [86]. Dehydrated SEs in some species demonstrated high plantlet recovery rates even after prolonged storage periods, up to 90% after 42 months of storage at 4 °C in grapevine, while in cassava, SSEs dehydrated to a 50% moisture content can be preserved at 16 °C for up to 12 months [86]. In other species, like alfalfa, desiccation reduced the embryo capacity to form SSEs, and only a short desiccation period of 10 days gave comparable results to fresh samples, making this storage method less suitable for germplasm conservation in alfalfa [103]. The inability to conserve the cocoa (*T. cacao*) germplasm via seed storage, and the vulnerability of field collections, made the development of an encapsulation–dehydration-based cryopreservation system a priority, and a developed system of SSE regeneration from cotyledonary epidermal cells without the callus phase proved to be of high genetic fidelity and therefore suitable for long-term conservation of this species [41].

In addition to its numerous applications in plant biotechnology, it is worth noting that studying SSE also offers tools for investigating fundamental plant processes, like morphological, physiological, and molecular aspects of embryogenesis in higher plants as well as mechanisms underlying cellular totipotency [5]. SSE is also a convenient way to study embryo development, offering many similarities between zygotic and somatic embryos at the morphological or molecular level [78]. Especially interesting is the ability of fully differentiated stomatal guard cells of PSEs to produce SSEs directly, providing a model for studying the totipotency and embryogenetic capacity of specialized somatic cells [61].

## 6. Challenges and Future Directions–Maximizing the Potential of Cyclic SE

Secondary or cyclic SE is a powerful tool in both plant biotechnology and fundamental biology research, yet its widespread application faces several challenges, which will have to be the focus of future research in order to maximize its potential.

Genotype dependence in many species remains an important challenge. Some genotypes inherently possess a high embryogenic potential that persists over long periods and many cycles, as demonstrated for *M. truncatula* [27]. The same study established that even the chosen developmental pathway, direct or indirect SSE, or the morphological differences observed among SSEs, can be influenced by the genotype effect [27]. The origin of these differences is still not well understood, but they are likely related to complex genetic and epigenetic factors that influence embryogenic competence. Nevertheless, the increased embryogenic potential of SSE compared to PSE can be manifested even in the less productive genotypes [78,94].

For many species, the embryo quality, maturation, and conversion potential are still problematic [48]. Physiological and morphological abnormalities and asynchrony in embryo development can be obstacles for wider commercial application. These abnormalities can affect SE germination and conversion to plantlets [36], but can be efficiently recycled through SSE [67]. Future efforts to reduce abnormalities and improve synchronicity would make the process more predictable and efficient for downstream applications [25]. Asynchrony in embryo development, where various developmental stages are present at the same time in culture, is a major constraint for the commercial application of this technology [71], but can be utilized in cyclic culture by, for example, separating cotyledonary SEs for germination, and using leftover embryogenic material for starting a new cycle.

Declining embryogenic potential due to aging or subculturing effects is a problem that largely affects most regeneration efforts, and still more research is needed to identify factors that can be modified to reverse these effects and prolong the embryogenicity of cultures. SSE provides a method, at least in some species, to restore or reinvigorate the embryogenic potential of aging/failing lines that show a diminishing maturation ability or produce abnormal embryos, especially for clones proven to be highly productive and valuable [78]. However, more research into the factors that control the number of cycles that can be maintained in culture is needed. Short-term PGR exposure during induction, or cycling between PGR exposure and elimination, could prove advantageous for prolonging embryogenic potential and reduce aging effects.

While SSE can achieve rapid multiplication, the initial induction of PSE can be labor-intensive and time-consuming, and it may not yield the expected results across diverse plant species. It can also be cost-ineffective for species easily multiplied by other methods, especially if those methods do not lead to a significantly higher rate of somaclonal variation [6,51].

Since the genetic and epigenetic aspect of SSE is still underexplored and many underlying mechanisms that regulate the expression of the SE program, direct or indirect pathway, or the ability to maintain the embryogenic potential in several cycles are largely unknown, more targeted molecular and histological analyses are required for the advancement of this field. Modern molecular techniques, including expression profiling, miRNA and lncRNA research, and profiling of methylation and other epigenetic differences, will prove invaluable for future SE research. The use of CRISPR/Cas and similar genome editing methods, to discover essential genes and signaling pathways, will facilitate advancement in this field. Additionally, more recent applications of CRISPR, including modulating methylation patterns or selective gene expression activation [104], open up even more horizons in SE research, possibly providing important insights into the role of epigenetic modifications in SSE. The ability of CRISPR/Cas to produce non-transgenic plants in the editing process is of particular importance in the context of potential commercial application of new superior varieties [105].

While extensive research has been conducted to elucidate the molecular mechanisms underlying PSE, molecular analyses related to SSE remain extremely rare. Instead, research on SSE is mainly focused on optimizing nutritional and culture conditions for embryo induction and maturation, which can considerably vary even within a single species. In many economically important species, the protocols for SE induction are often developed separately for each cultivar or clone, resulting in protocol redundancy and inconsistency. Therefore, future SSE studies should focus more on molecular analyses, integrating omics approaches, as understanding the underlying regulatory mechanisms will facilitate protocol standardization, which is particularly important for economically valuable species and will support more efficient propagation through SSE.

## 7. Conclusions

Secondary somatic embryogenesis is a versatile method in both fundamental plant biology and biotechnology, enabling efficient mass clonal propagation, genetic transformation, crop improvement, and germplasm conservation. SSE can proceed via direct or indirect developmental pathways, each with distinct advantages. Indirect SSE is typically more proliferative and preferred for large-scale propagation, but the callus phase increases the risk of somaclonal variation and genomic instability. Direct SSE, although less frequent and yielding fewer embryos, avoids the callus phase, produces embryos faster, and reduces genetic variation, which is particularly suitable for transformation experiments. Histological studies reveal that the cellular origin of SSE varies among species, from unicellular to multicellular. A unicellular origin is particularly useful for transformation, enabling a somatic embryo from a single transformed cell, while multicellular origins often require additional cycles to reduce chimerism. Abnormal embryo morphologies, including fused or multiple cotyledons and trumpet- or plate-like structures, remain common challenges, often caused by imbalanced culture conditions such as prolonged exposure to growth regulators, mainly synthetic auxin 2,4-D, which in higher concentrations, usually leads to malformed embryos. Since such abnormalities often negatively affect embryo germination and successful plantlet conversion, besides optimizing nutrient components, it is essential to develop other mitigation strategies, such as recycling abnormal embryos through SSE. The cyclic nature of SE enables the embryogenic potential to be maintained over successive cycles, significantly increasing the embryo yield and producing true-to-type plants, independent of the original explant. However, its efficiency varies among species, and physiological decline or accumulated genetic and epigenetic changes can limit the number of cycles. In addition to genetic stability, some of the major current challenges in this field relate to genotype dependence, embryo quality, maturation, and conversion potential. By integrating optimized culture conditions with modern molecular techniques such as omics approaches, methylation profiling, and genome editing tools such as CRISPR/Cas, SSE can be more reliably utilized for large-scale propagation, conservation, and transformation, maximizing its potential across various industries.

## Data Availability

The list of 133 references narrowly focused on secondary somatic embryogenesis (SSE) in plants, with the abstract, year of publication, and DOI downloaded from Scopus; the list is openly available in the RADaR-Digital Repository of Archived Publications Institute for Biological Research “Sinisa Stankovic” at https://hdl.handle.net/21.15107/rcub_ibiss_7767 (accessed on 6 November 2025).

## Abbreviations

The following abbreviations are used in this manuscript:

SE	Somatic embryogenesis
PSE	Primary somatic embryogenesis
SEs	Somatic embryos
PSEs	Primary somatic embryos
SSE	Secondary somatic embryogenesis
PGRs	Plant growth regulators
2,4-D	2,4-dichlorophenoxyacetic acid
SSEs	Secondary somatic embryos
MS	Murashige and Skoog medium
CIM	Callus induction medium
SH	Schenk and Hildebrandt medium
BA/BAP	Benzyladenine/Benzylaminopurine
NAA	1-naphthaleneacetic acid
CPPU	N-2-Chloro-4-pyridyl-N’-phenylurea
TIS	Temporary immersion system
